# A survey study of Chinese adolescents’ mental and interpersonal quality: Evidence from COVID-19 pandemic

**DOI:** 10.3389/fpsyg.2022.1049077

**Published:** 2022-10-24

**Authors:** Leping Huang, Yingfu Zhu, Wei Kang, Chunmu Zhu

**Affiliations:** ^1^Faculty of Psychology, Tianjin Normal University, Tianjin, China; ^2^School of Foreign Languages, Tianjin University of Commerce, Tianjin, China; ^3^Faculty of Medicine, University of Tsukuba, Tsukuba, Japan

**Keywords:** COVID-19, adolescents, adaptation status, interpersonal quality, grade differences

## Abstract

Since 2019, the COVID-19 pandemic, as a global public health emergency, has led to stringency measures (such as lockdown) of various degrees worldwide. As these measures such as social distancing measures and mandatory lockdown are intended to minimize social mobility, they have exerted remarkable impact on individuals’ mental health, particularly, adolescents and children. The mental health problems caused include fear, anxiety, sense of isolation and development of more maladaptive behaviors due to prolonged lockdown and restricted interpersonal contact. However, well adaption status and stable interpersonal relationships play an important role in maintaining and promoting the mental health of adolescents and children. This study aims to gain a new sight of understanding of the mental health quality of Chinese adolescents during the COVID-19 Pandemic in terms of adaptation and interpersonal quality. The study is based on a survey on a total of 7,318 junior and senior high school students aged 12–18 years in various regions of China, and intended to examine the differences in adolescent mental health quality by sociodemographic variables such as gender, grade, urban and rural areas, only child, and parental education level. Our study finds that Chinese adolescents show an overall good adaptation and interpersonal quality during the lockdown. However there exists disparity across different categories in developmental patterns of adaptation and interpersonal quality. In addition, good family environment, moderate financial and emotional support, higher parental education level, etc. contribute to the cultivation and improvement of adolescents’ mental health quality. Finally, we suggest that the government and researchers should pay more attention to adolescents’ mental health issues in terms of adaption status and interpersonal relationships during the COVID-19 pandemic, especially for disadvantaged social groups such as girls, younger students, and low-income family students.

## Introduction

During COVID-19 pandemic, governments adopt strict anti-virus measures and restrict human contact to avoid the spread of the virus (Fang et al., 2021; Galli et al., 2022), but this had a very negative impact on the mental health and behavior of individuals (Conti et al., 2020; Velavan and Meyer, 2020; Panda et al., 2021). Studies have found that after isolation and lockdown, children between the ages of 1.5 and 18 are more likely to experience irritability, obsessive–compulsive disorder, post-traumatic stress disorder, and thinking problems among others (Conti et al., 2020). It has also been reported that children who lack outdoor activities and opportunities to interact with their peers feel fearful, anxious, isolated, and develop more maladaptive behaviors due to the prolonged closure of public places such as schools, parks, and theaters (Duan et al., 2020; O’Sullivan et al., 2020; Yeasmin et al., 2020). This has a serious impact on their schooling and daily life, and can even lead to the development of mental illness and pose security risks to social development (Czeisler et al., 2020). Primary and secondary school is a critical period of growth and development for individuals who experience a series of significant physiological and psychological changes, and mental health is as important as physical health. Studies have shown that anxiety, depression, irritability, learning difficulties, adjustment disorders in interaction, and difficulties in adapting to the social environment are among the various common behavioral and psychological problems during COVID-19 pandemic (Colizzi et al., 2020; Duan et al., 2020). These problems also partly reflect the huge impact of the pandemic on the psychological growth of adolescents in both China and other countries (Cao et al., 2020; Husky et al., 2020; Charbonnier et al., 2022).

WHO defines health as “complete physical, mental, and social well-being, not merely negatively as the absence of disease or infirmity” (Grad, 2002). The mental health of adolescents is closely related to good adaption status and stable interpersonal relationships (Killgore et al., 2020; Wang et al., 2021). These intrinsic and relatively stable psychological qualities, formed by a combination of genetic and environmental factors, influence or determine the social, psychological, and physiological functions of individuals, which in turn affects their mental health status.

Therefore, the measures such as social distancing and mandatory lockdown have exerted remarkable impact on Chinese adolescents’ mental health. This paper aims to give a new sight of the mental health quality of Chinese adolescents during the COVID-19 pandemic in terms of adaptation and interpersonal quality. It is based on a survey on a total of 7,318 junior and senior high school students aged 12–18 years in various regions of China, and intended to examine the differences in adolescent mental health quality by sociodemographic variables such as gender, grade, urban and rural areas, only child, and parental education level. COVID-19 is spreading rapidly worldwide starting in late 2019 (Lima et al., 2020). As the case and severity of COVID-19 pandemic increase, mental health issues in adolescents need to be given high priority along with psychosocial support for patients and medical personnel (Xiong et al., 2022). Considered a survey on a total of 7,318 junior and senior high school students aged 12–18 years in various regions of China, this study intends to examine the differences in adolescent mental health quality by sociodemographic variables such as gender, grade, urban and rural areas, only child, and parental education level.

In addition, with regard to education policy, the issue of health care and reducing the burden of studies for China’s adolescents and children has been repeatedly urged and emphasized. Through documents such as the policy of ease the burden of excessive homework and off-campus tutoring for students undergoing compulsory education, the policy is clear in terms of reducing the burden in and outside school, reducing the burden of examination, evaluation, homework, and regulating the use of electronic products, etc., and the need to care for the health of primary and secondary school students in terms of eyesight, sleep, and body has been pointed out, urging schools and parents to raise awareness of students’ physical and mental health development. However, with the education disruptions and uncertain future during the pandemic, a large number of students reported experiencing various problems, such as studies troubles, emotional frustrations, and conflicts between parents and children among others (Lee, 2020).

The psychological state of students has been affected by the changes in society and the times. However, there are few studies on the impact of the COVID-19 epidemic and its related control measures on the mental health quality of adolescents. Therefore, in the context of COVID-19 pandemic and the policy of ease the burden of excessive homework and off-campus tutoring for students undergoing compulsory education, it is important to investigate the latest situation of the adaption status and interpersonal quality of Chinese adolescents, understand their trend, and analyze their influencing factors to more effectively carry out mental health education that is in line with the development characteristics of the times and the actual needs, and eliminate as much as possible the negative psychological and emotional influences, so that students’ physical and mental health can be promoted.

## Materials and methods

### Respondents

From April 1st to 30th, 2022, a total of 8,013 questionnaires were distributed to junior and senior high school students in China’s East and North regions using cluster sampling, and 7,318 valid questionnaires were obtained, with an effective rate of 91.3%. To select these regions, Chinese adolescents in these areas are well educated and participate in rich extracurricular activities. Their mental and interpersonal quality after the suddenly epidemic are more enlightening. More, these regions are relatively developed in China’s economy, which provided more communications for Chinese adolescents. Among them, 3,351 (45.79%) were male students and 3,967 (54.21%) were female students; 4,258 (58.19%) were junior high school students (19.20% in the first year, 20.59% in the second year and 18.40% in the third year) and 3,060 (41.81%) were senior high school students (20.11% in the first year and 21.70% in the second year); 4,017 (54.89%) were rural students and 3,301 (45.11%) were urban students; 2,625 (35.87%) were only child, 4,693 (64.13%) were non-only child. The 7,318 junior and senior high school students ranged in age from 12 to 18 years, with the average age of 15.37 ± 1.46 years, no history of neurological or mental disorders, and no pervasive developmental disorders. All tested adolescents signed an informed consent form before took the test.

### Tools

Our study uses Adaptation Scale for Adolescent (ASA). ASA contains six dimensions, including biological adaptation, emotional adaptation, interpersonal adaptation, study adaptation, social adaptation, and living adaptation, with a total of 22 questions. The higher the ASA score, the easier the individual is to adapt to the environment and physical and mental changes, i.e., the better the adaptation status. In this study, the Cronbach’s alpha coefficient for this scale was 0.95.

The study also uses Interpersonal Communication Scale (ICS). ICS contains three dimensions, including the ability of communication, interpersonal monitor, and interpersonal perception, with 16 questions. Scale items are scored from 1 point (not at all) to 5 point (fully), and the scores of each dimension are summed and averaged as the dimension score; the total average score is calculated by summing the scores of all items. The higher the ICS score, the better the individual is at interpersonal communication, the more he/she is able to perceive and experience the needs of others in communication, and more prominent the initiative and effect at interacting with objects. In this study, the Cronbach’s alpha coefficient for this scale was 0.94.

### Statistical processing

Excel was used to enter and organize the data. Subsequently, SPSS 23.0 software was used for statistical processing of the data. Statistical methods such as descriptive statistics, t test and analysis of variance were mainly used to analyze the data. Differences were considered statistically significant at *p* < 0.05.

## Results

### Characteristics of the adaptation status of Chinese adolescents

**Figure 1:**
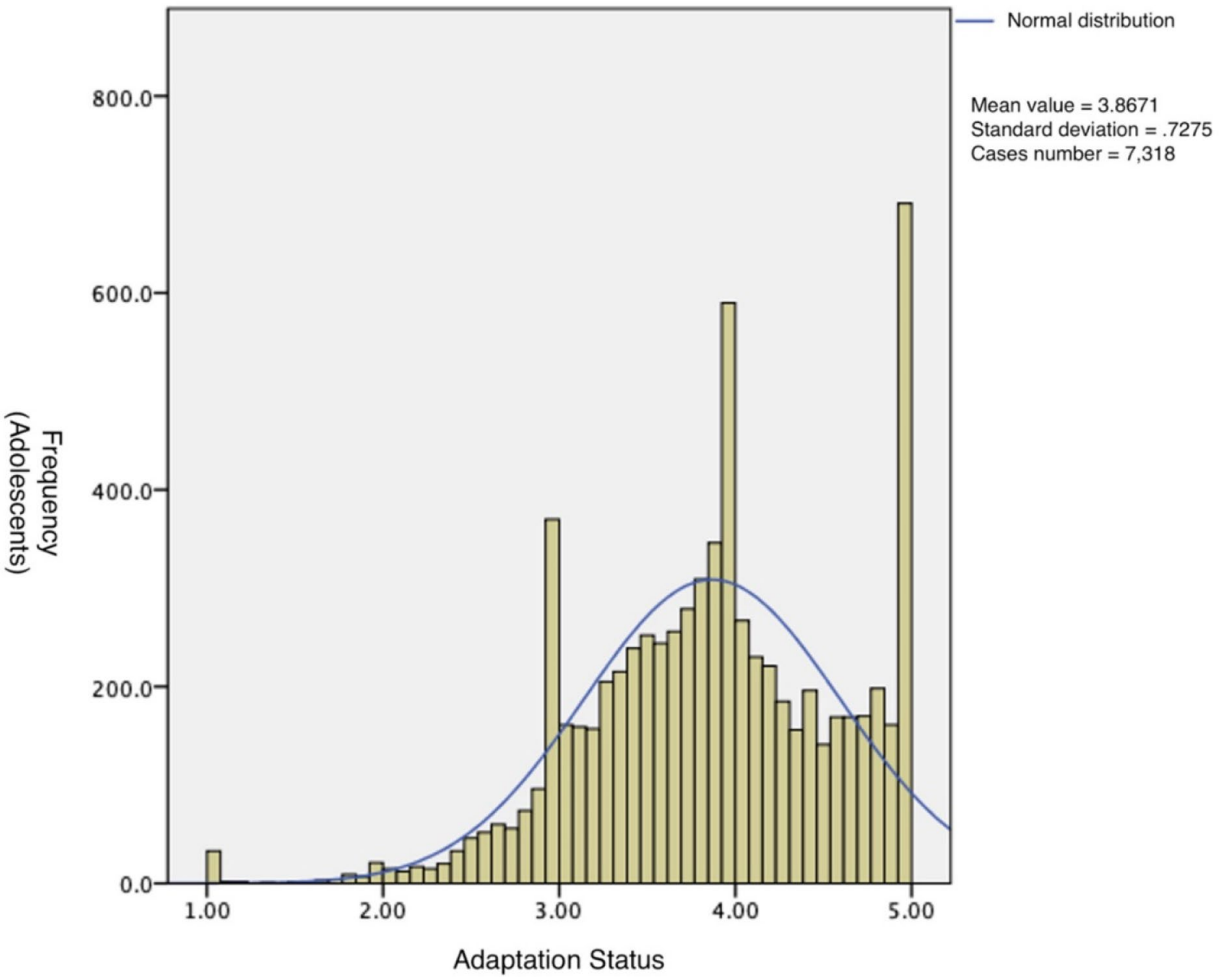
Adaptation status of Chinese adolescents. Horizontal coordinates describe the frequency of adolescents. And, the vertical coordinates describe the adaptation status.

Figure 1 Frequency distribution of Chinese adolescents on adaptation scores.

The data of 7,318 adolescents who participated in the survey were analyzed, and the results showed that the mean of the adaptation score of Chinese adolescents was 3.87, the standard deviation was 0.73, and the 95% confidence interval of the overall mean was 3.85 to 3.88. Data distribution was in line with normal distribution, with a skewness coefficient of-0.43. The skewness coefficient is less than 0; that is, when the heavy tail is on the left, the distribution is biased to the left. And, a kurtosis coefficient of 0.36. The kurtosis is greater than 0, the overall data distribution is steep compared with the normal distribution, which is a peak. The results are shown in Figure 1.

### Characteristics of interpersonal quality of Chinese adolescents

**Figure 2:**
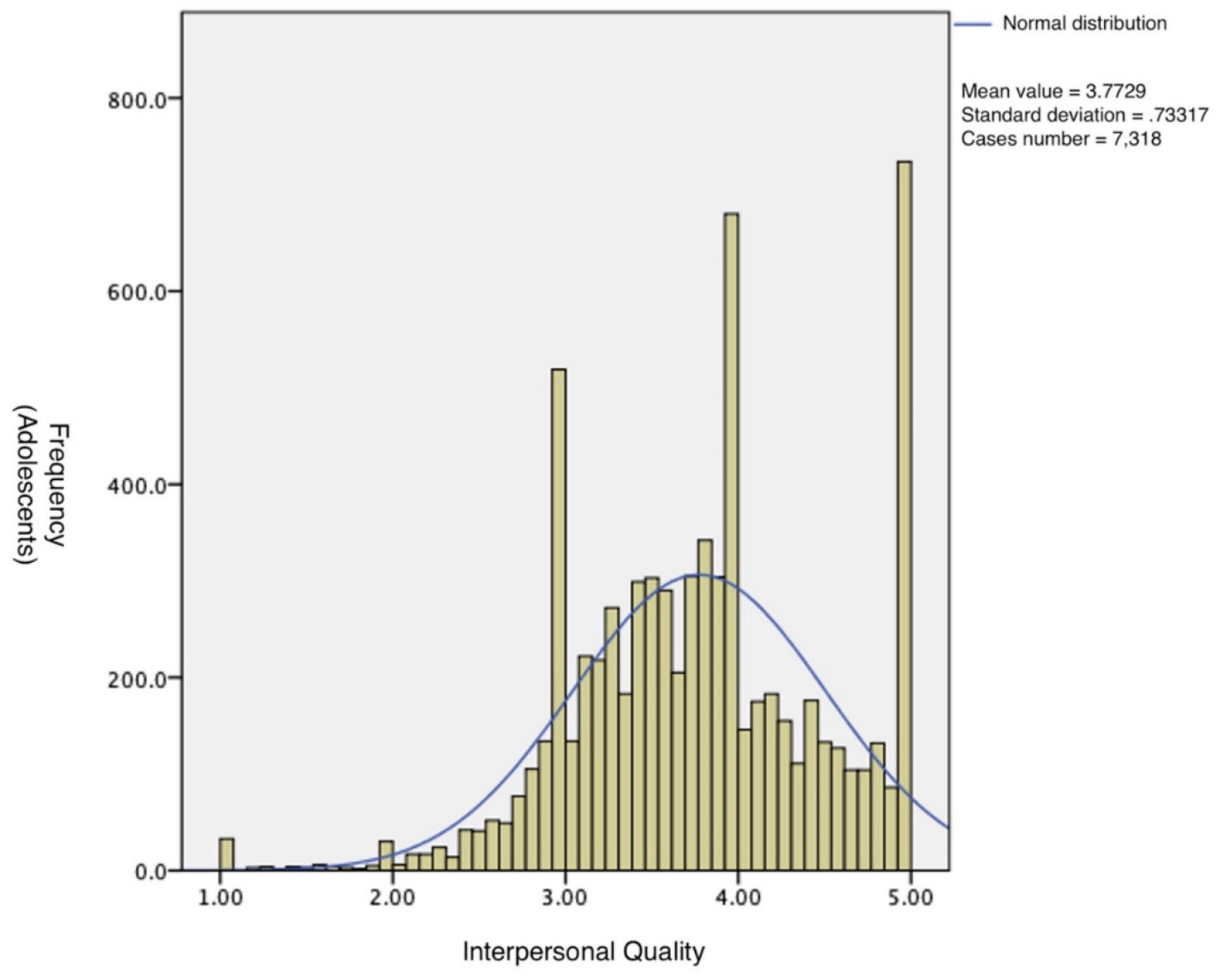
Interpersonal quality of Chinese adolescents. Horizontal coordinates describe the frequency of adolescents. And, the vertical coordinates describe the interpersonal quality.

Figure 2 Frequency distribution of Chinese adolescents on interpersonal quality scores.

The data of 7,318 adolescents who participated in the survey were analyzed, and the results showed that the mean of the adaptation score of Chinese adolescents was 3.77, the standard deviation was 0.73, and the 95% confidence interval of the overall mean was 3.76 to 3.79. Data distribution conformed to a normal distribution, with a skewness coefficient of-0.22. The skewness coefficient is less than 0; that is, when the heavy tail is on the left, the distribution is biased to the left. And, a kurtosis coefficient of 0.25. The kurtosis is greater than 0, the overall data distribution is steep compared with the normal distribution, which is a peak. The results are shown in Figure 2.

### Analysis of the trend of adaptation status and interpersonal quality of Chinese adolescents

One-way ANOVA results showed significant differences in overall scores across grades on ASA (*F* = 6.06, *p* < 0.001), but not on the ICS (*F* = 0.90, *p* > 0.05). Further analysis of the dimensions of each scale revealed that there was a grade effect (*p* < 0.05) for all dimensions except emotional adaptation on the ASA and interpersonal monitor on the ICS. As can be seen from Table 1, in terms of biological and study adaptation, the scores were highest in junior one students, gradually decreasing as the grade went up, lowest in junior three, rebounding in the first year of senior high school, but decreasing again after that; in terms of interpersonal, social, and living adaptation, the highest scores were obtained in junior one, gradually decreasing as the grade increased, the lowest scores were obtained in junior third, and the trend of recovery was observed in the first and second years of senior high school; as to the ability of communication, the highest score was obtained in junior one, gradually decreasing as the grade increased, the lowest score was obtained in junior three, and the recovery trend was shown in the first and second years of senior high school; the score of interpersonal perception was the lowest in junior one, increasing in two, and decreasing in junior three, and the highest score was obtained in senior one, but decreasing trend was observed after that.

**Table 1:** Trends in adaptation and interpersonal quality of Chinese adolescents in post-epidemic era.

Variables	Dimension	Junior one (*n* = 1,405)	Junior two (*n* = 1,507)	Junior three (n = 1,346)	Senior one (*n* = 1,472)	Senior two (*n* = 1,588)	*F* value	*p* value
Adaptation status	Biological adaptation	3.89 ± 1.01	3.86 ± 0.99	3.75 ± 1.07	3.90 ± 0.96	3.87 ± 0.96	4.88	0.001
	Emotional adaptation	3.84 ± 0.85	3.78 ± 0.89	3.76 ± 0.88	3.79 ± 0.85	3.78 ± 0.81	1.59	0.175
	Interpersonal adaptation	3.89 ± 0.83	3.83 ± 0.87	3.76 ± 0.89	3.82 ± 0.82	3.86 ± 0.80	4.34	0.002
	Study adaptation	3.95 ± 0.84	3.81 ± 0.91	3.76 ± 0.91	3.77 ± 0.87	3.76 ± 0.87	12.31	0.000
	Social adaptation	3.94 ± 0.85	3.92 ± 0.85	3.85 ± 0.88	3.89 ± 0.83	3.91 ± 0.80	2.47	0.042
	Living adaptation	3.96 ± 0.86	3.86 ± 0.89	3.82 ± 0.90	3.85 ± 0.87	3.86 ± 0.86	5.20	0.000
	Overall score	3.94 ± 0.70	3.87 ± 0.73	3.80 ± 0.77	3.86 ± 0.73	3.87 ± 0.70	6.06	0.000
Interpersonal quality	Ability of communication	3.77 ± 0.87	3.67 ± 0.94	3.65 ± 0.95	3.67 ± 0.93	3.67 ± 0.88	3.65	0.006
	Interpersonal monitor	3.99 ± 0.75	4.00 ± 0.76	3.96 ± 0.80	3.97 ± 0.74	3.99 ± 0.72	0.78	0.541
	Interpersonal perception	3.57 ± 0.88	3.66 ± 0.89	3.61 ± 0.90	3.71 ± 0.83	3.70 ± 0.82	6.52	0.000
	Overall *score*	3.78 ± 0.71	3.78 ± 0.74	3.74 ± 0.78	3.78 ± 0.73	3.79 ± 0.70	0.90	0.460

### Analysis of adaptation status and interpersonal quality differences among Chinese adolescents

According to the analysis results, male students scored significantly higher than female students on both ASA and ICS (*p* < 0.001), urban students were higher than rural students (*p* < 0.001), only children were higher than non-only children (*p* < 0.001), and statistically significant differences existed between the scores of students with different levels of parental education (*p* < 0.001).

In terms of adaptation status and interpersonal qualities, students with fathers’ education level of junior high school and below scored significantly lower (*p* < 0.05) than students with fathers’ education level of senior high school, specialized secondary school, junior college, Bachelor, Master and above; students with mothers’ education level of junior high school and below scored significantly lower than students with mothers’ education level of senior high school, specialized secondary school, junior college, and Bachelor (*p* < 0.001) (see Table 2).

**Table 2:** Comparison in adaptation and interpersonal quality among Chinese adolescents (M ± SD).

Variables	Classification	Adaptation status	Interpersonal qualities
Gender	Male (*n* = 3,351)	3.93 ± 0.76	3.82 ± 0.77
	Female (*n* = 3,967)	3.81 ± 0.70	3.73 ± 0.69
	*t* value	−6.68	−5.18
	*P* value	0.00	0.00
Urban and rural	City (*n* = 3,301)	3.94 ± 0.71	3.84 ± 0.71
	Rural (*n* = 4,017)	3.81 ± 0.74	3.72 ± 0.75
	*t* value	8.03	7.16
	*P* value	0.00	0.00
Only child	Yes (*n* = 2,625)	3.93 ± 0.71	3.82 ± 0.73
	No (*n* = 4,693)	3.83 ± 0.73	3.75 ± 0.74
	*t* value	−5.22	−3.78
	*P* value	0.00	0.00
Father’s education level	Junior high school and below (*n* = 3,851)	3.78 ± 0.73	3.70 ± 0.75
	Senior high school and specialized secondary school (*n* = 2,035)	3.93 ± 0.72	3.85 ± 0.71
	Junior college (*n* = 775)	4.01 ± 0.67	3.86 ± 0.70
	Bachelor (*n* = 585)	4.00 ± 0.71	3.86 ± 0.70
	Master and above (*n* = 72)	4.04 ± 0.75	3.96 ± 0.78
	*F* value	32.28	20.83
	*p* value	0.00	0.00
Mother’s education level	Junior high School and Below (*n* = 4,239)	3.79 ± 0.73	3.70 ± 0.73
	Senior high school and Specialized Secondary School (*n* = 1,776)	3.95 ± 0.73	3.86 ± 0.74
	Junior college (*n* = 699)	4.03 ± 0.66	3.88 ± 0.69
	Bachelor (*n* = 545)	4.02 ± 0.67	3.87 ± 0.69
	Master and above (*n* = 59)	3.86 ± 0.86	3.84 ± 0.85
	*F* value	34.36	22.13
	*p* value	0.00	0.00

## Discussion

The study result showed that Chinese adolescents performed better in terms of adaptation status and interpersonal quality during the COVID-19 pandemic, with mean scores above 3, and their overall level was high. This indicates that, when facing various problems in school, life, and society brought about by major public health emergencies, Chinese adolescents are able to flexibly adopt strategies and methods to positively adapt and solve them. On the one hand, most of the respondents are located in East and North China, which have large populations, better economic development, abundant medical resources and a complete medical system, where epidemics can be effectively controlled and prevented, thus greatly enhances the sense of security and well-being of adolescents in these regions. On the other hand, the policies set forth and implemented by the Chinese government provide unique opportunities and conditions for youth to highly develop their adaptive and interpersonal skills, which have a positive impact on the mental health quality of adolescents. For example, Chinese teachers usually play melodic and soothing music after school online to shape students’ values and relieve their stress, and encourage regular and moderate exercise at home, which not only improves physical function and health, but also regulates individual emotions and improves poor mood and psychology (Lin and Xu, 2020). Therefore, during the pandemic, Chinese adolescents showed good performance in terms of adaptive skills, social interactions, and building good interpersonal relationships.

Chinese adolescents’ scores on most dimensions of adaptation show a trend of higher scores from the first year of junior high school, decreasing as grade increases, and increasing in senior one and two, which is consistent with previous studies (Zhou et al., 2020). This may be due to the fact that learning tasks and social life are relatively simple in junior high school, and there are fewer psychological conflicts and contradictions, so students have a good self-perception of their adaptation status in all aspects. However, as students grow older, the physiological changes in adolescent development, the increase of studies pressure, and the complexity of interpersonal relationships, psychological conflicts and contradictions arise constantly, and students may experience some degree of maladaptation in various aspects (Jin et al., 2022). The contrast between their independent self-consciousness and immature psychological development also leads to swings in interpersonal development. In addition, students at this stage are mainly devoted to intense studying, and the pressure of studies and further education may lead to a downward trend as they do not have sufficient time to consciously experience and enhance interpersonal relationships, limiting their opportunities for interpersonal interaction. Lin and Li (2005) found that students in the first and second years of senior high school have entered the mid-youth stage, as individuals’ thinking (cognitive, intellectual) and morals (sociality) are gradually stabilizing, physiological and psychological development also tend to mature, personality has been basically set, lifestyle basically tends to be fixed and habitual, adaption status and social skills increase, and therefore, tend to be stable and rebound in terms of adaptive status and interpersonal quality. It is clear from this that adaptation in the lower grades is not at the same level of that in the upper grades, i.e., adaptation in the lower grades is only at a low level, while adaptation in the upper grades is at a high level. However, this study was not able to select college students as subjects, so further research is needed to support this conclusion.

This study also found that male students scored significantly higher than female students on both ASA and ICS.

Compared with rural students, urban students have better living conditions and educational resources, a good family environment, and their parents pay more attention to children’s growth and education. Students from only child families receive more financial and emotional support in their studies and lives, and their adaptive skills and interpersonal relationships are better than those of students from non-only child families in all aspects. This is consistent with related research (Magnuson and Duncan, 2006). Rural students will be under more financial and psychological pressure, and, due to their limited family conditions and high studies and living expenses, most rural students will choose to earn their living expenses through tutoring and work-study program, which will increase their learning and social pressure at the same time. However, such students may be in financial difficulties due to the epidemic (Fang et al., 2022). Multiple stresses may contribute to rural students’ mental health problems in adaptation, interpersonal communication among others. This is also true for students from non-only child families. Parents with higher education levels are more adaptable and socially competent, more optimistic in dealing with problems (Xiao et al., 2020), and more concerned about all aspects of their children’s development and education, thus playing an important role in their children’s growth, through genetic factors and by personal example as well as verbal instruction. Meanwhile, well-educated parents usually have higher socio-economic status, better material base and more social experience (Sánchez-Rodríguez et al., 2022), and are able to create a better learning and living environment for their children, especially with a reasonable education, which is conducive to their children’s physical and mental growth (Cao et al., 2020). For example, during the COVID-19 pandemic, these parents engage their children in online classes, online interactions, and video games by means of smartphones and other digital technologies to enhance their children’s studies motivation and well-being (Limone and Toto, 2022), and these parents strictly control their children’s use of smartphones and other digital technologies to prevent children from developing negative emotional and psychological problems due to excessive dependence on digital devices (Ravens-Sieberer et al., 2021; Vuorre et al., 2021). This suggests that, during the COVID-19 pandemic, a good family environment, moderate financial and emotional support, and higher parental education level, among others, contribute to the cultivation and improvement of adolescents’ mental health qualities. Therefore, the government and schools should pay more attention to the psychological dynamics of vulnerable groups such as girls, lower grades students, poor students, and non-only child during the epidemic, and provide them with necessary economic support and mental health services.

## Limitations

First, this study lacks data from the pre-pandemic period and control groups in the longitudinal study. Second, the various assessment variables involved in the study were derived from subjects’ self-perception reports, and there may be a social desirability effect. Finally, only two variables of mental health quality, adaptive ability and interpersonal quality, were selected for analysis, and thus we could not provide a systematic and in-depth understanding of the overall mental health quality of Chinese adolescents during the pandemic. However, the pandemic is now continuing to worsen and may bring additional changes and problems to the minds and bodies of children and adolescents. Future studies could conduct further longitudinal studies to expand the sample source and cover more psychological variables to track the long-term effects of the COVID-19 pandemic on various aspects of children’s and adolescents’ mental health.

## Conclusion

In this paper, we analyzed and compared the adaptation status and interpersonal quality of Chinese adolescents in different grades and categories, and found that a series of restrictive measures during the COVID-19 epidemic had a certain degree of negative impact on Chinese adolescents’ mental health quality, but generally showed a good status. In addition, this study emphasizes the importance of a good family environment, moderate emotional communication, and peer support for adolescents’ mental health during the lockdown and isolation of the pandemic.

## Data availability statement

The raw data supporting the conclusions of this article will be made available by the authors, without undue reservation.

## Author contributions

LH and YZ: conceptualizing, writing, and drafting-original draft. WK: data and methodology. CZ: review and editing. All authors contributed to the article and approved the submitted version.

## Conflict of interest

The authors declare that the research was conducted in the absence of any commercial or financial relationships that could be construed as a potential conflict of interest.

## Publisher’s note

All claims expressed in this article are solely those of the authors and do not necessarily represent those of their affiliated organizations, or those of the publisher, the editors and the reviewers. Any product that may be evaluated in this article, or claim that may be made by its manufacturer, is not guaranteed or endorsed by the publisher.
